# Perturbations of the ileal mycobiota by necrotic enteritis in broiler chickens

**DOI:** 10.1186/s40104-021-00628-5

**Published:** 2021-10-09

**Authors:** Qing Yang, Jing Liu, Kelsy J. Robinson, Melanie A. Whitmore, Sydney N. Stewart, Guolong Zhang

**Affiliations:** 1 Department of Animal and Food Sciences, Oklahoma State University, Stillwater, OK USA; 2 Present address: Poultry Production and Product Safety Research Unit, USDA–Agricultural Research Service, Fayetteville, AR USA; 3 Present address: Safety and Security Division, Institute for Public Research, CNA, Arlington, VA USA

**Keywords:** Antimicrobial resistance, *C. perfringens*, Dysbiosis, ITS sequencing, Microbiome, Mycobiota, Necrotic enteritis, Poultry

## Abstract

**Background:**

Intestinal microbiota is critical for maintaining animal health and homeostasis. However, involvement of the fungal community, also known as the mycobiota, in animal health and disease is poorly understood. This study was aimed to examine the association between the intestinal mycobiota and the severity of necrotic enteritis (NE), an economically significant poultry disease.

**Methods:**

A total of 90 day-of-hatch Cobb broilers were infected with *Eimeria maxima* on d 10, followed by an oral challenge with *C. perfringens* on d 14 to induce NE, while another 10 broilers were served as mock-infected controls. On d 17, the lesions in the jejunum were scored, and the ileal digesta were subjected to DNA isolation and real-time PCR quantification of total bacterial and fungi populations. Internal transcribed spacer 2 (ITS2) amplicon sequencing was also performed to profile the ileal mycobiota composition. Changes in the ileal mycobiota in response to NE were investigated. Spearman correlation analysis was further conducted to identify the correlations between relative abundances of individual ileal fungi and the severity of NE.

**Results:**

While the total bacterial population in the ileum was increased by 2- to 3-fold in NE chickens, the total fungal population was progressively declined in more exacerbated NE, with the most severely infected chickens showing a nearly 50-fold reduction relative to mock-infected controls. Richness of the ileal mycobiota also tended to reduce in chickens with NE (*P* = 0.06). Compositionally, among 30 most abundant fungal amplicon sequence variants (ASVs), 11 were diminished and 7 were enriched (*P* < 0.05), while 12 remained largely unchanged in NE-afflicted chickens (*P* > 0.05). Multiple *Wallemia* and *Aspergillus* species were markedly diminished in NE (*P* < 0.05) and also showed a significant negative correlation with NE severity (*P* < 0.05).

**Conclusions:**

Dysbiosis of the ileal mycobiota is induced evidently by NE and the extent of the dysbiosis is positively correlated with disease severity. These findings suggest a possible role of the intestinal mycobiota in NE pathogenesis and highlight the mycobiota as a new potential target for NE mitigation in poultry.

**Supplementary Information:**

The online version contains supplementary material available at 10.1186/s40104-021-00628-5.

## Introduction

The gastrointestinal (GI) tract of humans and animals is populated with a diverse group of microbes known as the microbiota that include bacteria, fungi, archaea, protists, and viruses, with bacteria being the most predominant [1, 2]. The bacterial microbiota is well known to be critically involved in host physiology and immune development [1, 2]; however, the role of the fungal community, known as the mycobiota, that plays in health and diseases is less studied and understood. Recent studies have suggested that a healthy intestinal mycobiota appears to be important for maintaining host homeostasis, modulating host immune responses, and competitive exclusion of pathogens [3–5]. For example, colonization of *C. albicans* protects mice against infections of virulent fungi and bacteria by stimulating the expansion of Th17 cells, activating neutrophils, and thus enhancing host defense against extracellular pathogens in mice [6, 7]. Alterations in the intestinal mycobiota are also linked to exaggerated inflammation in diseases such as human inflammatory bowel disease (IBD) [8, 9], allergic airway diseases [10, 11], and colorectal cancer [12]. The interplay between the intestinal mycobiota and microbiota is critical for intestinal homeostasis [4]. Studies have shown disease-specific bacteria-fungi networks [8, 13], highlighting the significance of the fungal community in host health and underscoring a need for further investigation of the mycobiome.

Little is known about the intestinal mycobiota in poultry. We recently revealed the mycobiota in the upper GI tract to be more diverse than the mycobiota in the lower GI tract of chickens [14]. Unlike the intestinal bacterial microbiota, which appears to become stabilized between d 21–28, the cecal mycobiota remains unstable beyond d 28 [14]. A study of the turkey ileal mycobiota revealed a similar kinetic trend [15]. Furthermore, alternations in the ileal mycobiome are significantly correlated with the bacterial changes in response to a low-dose antibiotic and probiotics [15]. However, the involvement of the intestinal mycobiota in poultry diseases has not been studied to date. The intestinal mycobiota-microbiota interplay in the context of a disease remains unknown.

Necrotic enteritis (NE), caused by pathogenic Gram-positive bacterium *C. perfringens*, is one of the most common and economically devastating enteric diseases in poultry [16]. NE-induced disruption of the intestinal microbiota is well-documented [17]. Although the changes in the microbiota diversity vary among studies, the intestinal microbiota in NE chickens is generally characterized by an overgrowth of *C. perfringens* and *Escherichia*/*Shigella*, with a reduction of lactic acid bacteria (e.g. *Lactobacillus* and *Weissella*) and short-chain fatty acid (SCFA) producers (e.g. *Lachnospiraceae* species) [17]. However, the impact of NE on the intestinal mycobiota of chickens is currently unknown. The purposes of this study were to investigate the ileal mycobiota changes in response to NE in broiler chickens and further reveal a possible correlation between the mycobiota and disease severity, laying a foundation for potential development of the mycobiome-based approaches to mitigating NE in poultry.

## Materials and methods

### Chickens and co-infection model of NE

Non-vaccinated day-of-hatch male Cobb broiler chicks were obtained from Cobb-Vantress (Siloam Springs, AR), tagged individually with wing bands, and assigned randomly to floor pens with fresh wood shavings. Chicks were provided ad libitum with tap water and an antibiotic-free corn-soybean starter diet (crude protein 21.5%) that meets or exceeds the nutrient requirements of the National Research Council (NRC) recommendations (1994). The lighting schedule was set as 23 L:1D in the first week and 18 L:6D afterwards. The room temperature was maintained at 32 °C in the first week and reduced to 30 °C and 27 °C in the second and third week, respectively. All animal procedures were approved by the Institutional Animal Care and Use Committee at Oklahoma State University under the protocol number AG-16-10.

On d 10, a total of 100 chickens were individually weighed after overnight fasting and transferred to 17 battery cages with 5–6 animals per cage for experimental induction of NE as previously described [18, 19]. Upon transfer, 90 chickens in 15 cages were immediately challenged with 5 × 10^3^ sporulated oocysts of *E. maxima* strain M6 [20] in 1 mL saline via oral gavage, while the remaining 10 chickens in two cages were gavaged with 1 mL saline only and served as mock-infected controls. On d 14, after overnight fasting, 90 chickens that received *E. maxima* were orally inoculated again with approximately 4 × 10^8^ colony-forming unit (CFU) of *netB*- and *tpeL*-positive *C. perfringens* strain Brenda B [21] in 2 mL of overnight culture, which was prepared by sequential passage in cooked meat medium and fluid thioglycollate medium as described. Ten chickens in the mock-infected group were administrated with 2 mL fluid thioglycollate medium only.

All animals were monitored twice daily till d 17 for behavior and clinical signs. Mortalities were recorded daily and chickens reluctant to move were euthanized to alleviate undue pain. All surviving birds were weighed individually and euthanized through CO_2_ asphyxiation on d 17. Gross lesions of NE in the small intestine were evaluated in a blind manner using a 0–6 scoring system as proposed [18]. Briefly, the lesion scoring criteria were as follows: score 0 = no gross lesions, score 1 = thin or friable intestinal walls, score 2 = focal necrosis or ulceration (1–5 foci), score 3 = focal necrosis or ulceration (6–15 foci), score 4 = focal necrosis or ulceration (> 16 foci), score 5 = patches of 2- to 3-cm long necrosis, and score 6 = extensive necrosis typical of field cases. Contents from the proximal ileum (2–3 cm distal to Meckel’s diverticulum) were collected and stored at − 80 °C for microbial DNA extraction. Weight loss of infected chickens between d 10 and d 17 was calculated, relative to mock-infected controls.

### Microbial DNA extraction

Microbial genomic DNA of the ileal contents was extracted using the ZR Fecal DNA MicroPrep Kit (Zymo Research, Irvine, CA) following the manufacturer’s protocol. The resulting DNA concentration and purity were quantified using Nanodrop 1000 Spectrophotometer (Thermo Fisher Scientific, Wilmington, DE) and used subsequently for microbial quantification and fungal ITS2 amplicon sequencing.

### Quantification of total fungal and bacterial populations

Total populations of the fungi and bacteria in the ileal digesta were measured using Femto Fungal and Bacterial DNA Quantification Kits (ZYMO Research, Irvine, CA), respectively. Sample dilution and quantitative PCR (qPCR) were performed according to the manufacturer’s directions. Known quantities of purified genomic DNA of *Saccharomyces cerevisiae* and *Escherichia coli* were used to establish standard curves for fungi and bacteria, respectively. Total genome copies of the fungi or bacteria were estimated using the following formula as recommended by the manufacturer: genome copy number = DNA (g) / (g-to-bp constant × genome size), where the g-to-bp constant is 1.096 × 10^− 21^ g/bp and the average genome size of bacteria and fungi is 3.87 Mb [22] and 40 Mb [23], respectively. Results were expressed as fungal or bacterial genome copy number/g digesta, and the ratio of total genome copies of fungi to that of bacteria was further calculated for individual animals.

### Fungal ITS2 sequencing and bioinformatics

Microbial DNA of the ileal contents was subjected to ITS2 sequencing for the mycobiota profiling. The ITS2 region was amplified by PCR using the primers ITS3-2024F (5′-GCATCGATGAAGAACGCAGC-3′) and ITS4-2409R (5′-TCCTCCGCTTATTGATATGC-3′) [24]. The ITS2 amplicon library was constructed using the NEBNext® Ultra™ DNA Library Prep Kit (New England Biolabs, Ipswich, MA) and subsequently sequenced on an Illumina HiSeq platform by Novogene (Beijing, China). PE250 paired-end reads were then processed using QIIME 2 v.2019.10 [25]. After demultiplexing and removal of adapters, sequence reads were denoised using Deblur [26] to generate amplicon sequence variants (ASVs). Taxonomic classification of fungal ASVs was implemented using Naive Bayes classifiers against the UNITE reference database (v.8.2). The taxonomies of top 30 and NE-correlated fungal ASVs were confirmed by BLAST against the NCBI nucleotide database. The ASVs present in less than 5% of chickens were excluded from the analysis. The mycobiome sequencing data was normalized using the cumulative-sum scaling method in the R ‘metagenomeSeq’ package to correct uneven sampling depths [27].

Alpha and beta diversities of the fungal community were computed with R ‘phyloseq’ package (v.1.30.0) [28]. The number of ASVs, Pielou’s evenness index, and Shannon index were used to indicate the richness, evenness, and overall alpha diversity, respectively, whereas beta diversity was determined using weighted and unweighted UniFrac distances [29]. The mycobiota composition was indicated as relative abundances of fungal taxa at phylum, family, genus, and ASV levels. Fungal ASVs present in at least 20% of chickens were subjected to the linear discriminate analysis (LDA) effect size (LEfSe) analysis [30] to identify differentially enriched fungi between healthy and mildly-infected chickens as well as between mild and severe NE, with the cutoff at *P* < 0.05 and LDA score > 2.0. Spearman rank correlation analysis was performed between relative abundances of the fungal taxa existing in > 20% of chickens and NE severity indicated by lesion scores and weight loss. Spearman correlation coefficient was computed using the corr.test function in R ‘psych’ package (v.1.9.12.31) and displayed in Heatmap using the ‘pheatmap’ package (v.1.0.12) in R. Fold changes in relative abundance of significant NE-correlated taxa were calculated relative to that of mock-infected healthy chickens. Furthermore, ileal fungi-bacteria correlation was performed based on the Spearman correlation. NE severity-correlated fungal and bacterial taxa common in > 20% of chickens were included in the correlation analysis. The correlation matrix was plotted with R ‘corrplot’ package (v.0.84). Additionally, the ‘ggplot2’ package (v.3.3.0) [31] was used to make graphs in R.

### Bacterial 16S rRNA gene sequencing and bioinformatics

Microbial DNA of the chicken ileal contents was also subjected to 16S rRNA gene sequencing for profiling the microbiota as previously described [32, 33]. The V4 region of the bacterial 16S rRNA gene was amplified by PCR using the primers 515F (GTGCCAGCMGCCGCGGTAA) and 806R (GGACTACHVGGGTWTCTAAT) and sequenced on Illumina MiSeq, processed with QIIME2, denoised with Deblur, and classified using the Greengenes database. The composition of the ileal microbiota was indicated by relative abundance of bacterial taxa at phylum, order, family, genus, and ASV levels. Those bacteria that were commonly present in > 20% of the samples were further calculated for their association with the lesion score using Spearman correlation analysis as described above for the mycobiome.

### Statistical analysis

Statistical analysis and visualization were achieved in GraphPad Prism 8 (GraphPad Software, La Jolla, CA) and RStudio (v.1.2.1578) (RStudio, Boston, MA). Statistical significance was measured using parametric or non-parametric methods, depending on the normality of data as determined by the Shapiro-Wilk test. Weight loss, total fungi or bacteria, and the fungal/bacterial ratio were subjected to one-way analysis of variance (ANOVA) and Tukey’s *post-hoc* test, while alpha diversity and fungal relative abundance among groups were compared using Kruskal-Wallis and pairwise Wilcoxon rank-sum tests. The significance of beta diversity was measured by permutational multivariate analysis of variance (PERMANOVA) with 999 permutations using the R ‘vegan’ package (v.2.5.6). In Spearman correlation, the false discovery rate (FDR) was controlled using the Benjamini-Hochberg procedure. *P <* 0.05 or FDR < 0.05 was considered statistically significant.

## Results

### Growth impairment and ileal fungal load reduction by NE

As expected, sequential infections of chickens with *E. maxima* and *C. perfringens* induced clinical symptoms of NE including lethargy, anorexia, and diarrhea. Among 90 chickens infected, 33 died or were euthanized due to NE illness by d 17. All surviving chickens were scored for the severity of intestinal lesions using a 0–6 scale of a scoring scheme [18]. While the intestines of all 10 mock-infected chickens were apparently healthy and received a score of 0, all infected chickens had intestinal abnormalities, with lesions occurring primarily in the jejunum and proximal ileum. Among all infected chickens that were survived, 15 were scored 1, and 17 were given a score of 2. Five chickens were scored 5, while 13 birds were scored 6. Two infected chickens with a score of 3 and another two with a score of 4 were not included in the analyses because of the small sample size.

**Fig. 1:**
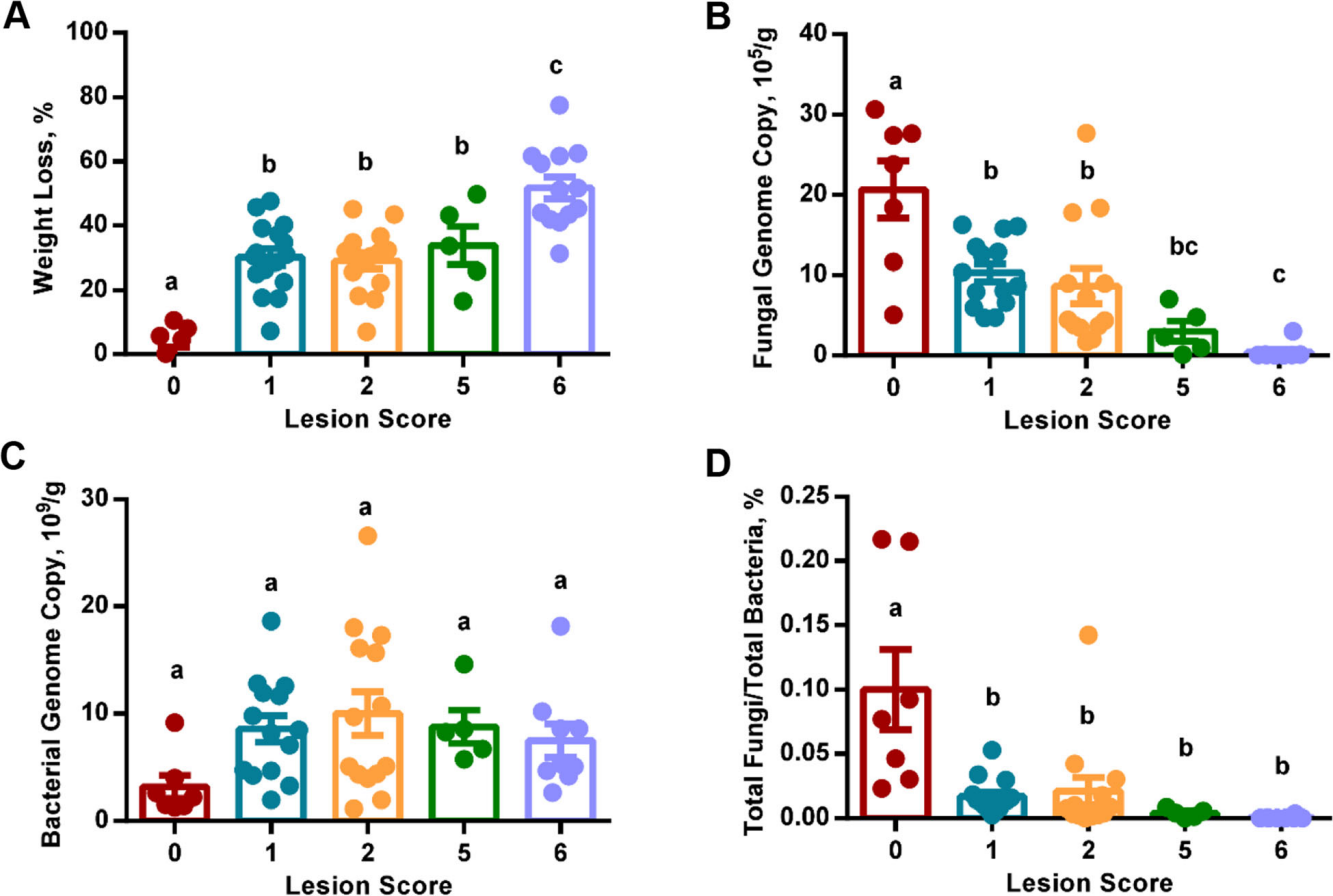
Weight loss and populations of the ileal fungi and bacteria in chickens with different severities of necrotic enteritis (NE)*.* Chickens were sequentially infected with *E. maxima* and *C. perfringens* to induce NE and separated into five groups based on their intestinal lesion scores. NE-induced weight loss (%) **A** was calculated relative to mock-infected controls. The total fungal genome copy numbers **B** and total bacterial genome copy numbers **C** per gram of the ileal contents were quantified using qPCR. The percentage (%) of total fungal genome copies relative to total bacterial genome copies **D** was also calculated. Results were expressed as means ± SEM and individual dots represented the results of individual animals. Statistical significance (*P* < 0.05), denoted by different superscripts, was determined using one-way ANOVA and Tukey’s *post-hoc* test

As a result, chickens were categorized into five groups (score-0, − 1, − 2, − 5, and − 6) based on their intestinal lesion scores. Although all groups of chickens had a similar body weight on d 10 prior to infection, weight loss of infected chickens was gradually increased as the lesions became more severe, with animals scored 1, 2, and 5 showing an approximately 30% weight loss as compared with mock-infected controls, while chickens scored 6 having a 52% reduction in weight gain between d 10–17 (Fig. 1A).

To evaluate the influence of NE on the intestinal microbial load, total fungal and bacterial populations in the ileal contents were quantified using qPCR. While mock-infected control (score-0) chickens had approximately 2 × 10^6^ of fungal genome copies/g digesta, infected chickens showed a significant progressive decrease (*P* < 0.05) with exacerbation of NE (Fig. 1B). Chickens with a score of 1 or 2 harbored approximately 1 × 10^6^ of fungal genome copies/g digesta; however, the fungal genomes were decreased to 3.0 × 10^5^/g digesta in chickens scored 5, which further declined to 4.3 × 10^4^/g digesta in severely infected chickens with a lesion score of 6 (Fig. 1B). By contrast, the genome copies of total bacteria were increased only by 2- to 3-fold in NE chickens, showing a plateau with chickens scored 2 (Fig. 1C). As a result, total fungal genome copies accounted for approximately 0.1% of total bacterial genome copies in the ileum of healthy broiler chickens, but was gradually declined in more severe NE (Fig. 1D). Total fungal population was decreased by approximately 5-fold to represent 0.02% of total bacteria in mildly infected chickens with a score of 1 or 2 and further reduced to only account for approximately 0.004% and 0.0006% of the bacterial population in more severe NE chickens with a lesion score of 5 and 6, respectively.

### Alternations in the diversity of the ileal mycobiota in NE

**Fig. 2:**
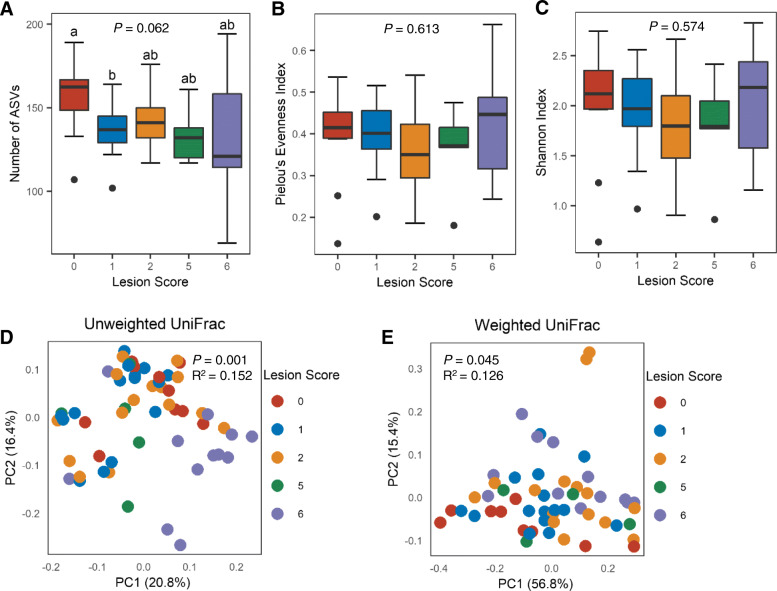
Alpha and beta diversities of the ileal mycobiota in healthy and NE chickens. Chickens were sequentially infected with *E. maxima* and *C. perfringens* to induce NE and separated into five groups based on their intestinal lesion scores. The number of amplicon sequence variants (ASVs) **A**, Peilou’s evenness index **B**, and Shannon index **C** were shown in box and whisker plots. Each box indicated median, 25th and 75th percentiles, while whiskers extended to 1.5 interquartile range. Significance was evaluated using Kruskal-Wallis test, while pairwise comparisons were implemented using Mann–Whitney U test. Different superscripts denoted significance (*P* < 0.05) in pairwise comparisons. Principal coordinates analysis (PCoA) plots were based on unweighted **D** and weighted UniFrac distances **E**. Each dot represented an individual ileal digesta sample. The *x* and *y* axes indicated the percentage of variation explained by two principal coordinates. Significance was determined using PERMANOVA

To investigate potential alternations of the intestinal mycobiome in NE, microbial DNA was isolated from the ileal digesta of both infected and mock-infected broilers and subjected to ITS2 sequencing. A total of 3,978,808 raw reads were obtained from 65 ileal digesta samples and analyzed using QIIME 2. After Deblur denoising, 1,512,578 sequence reads were left, with an average of 23,270 ± 1015 (SD) reads per sample, from which a total of 642 ASVs were generated. Samples with lesion scores of 3 and 4 were excluded from downstream analyses due to the small sample size. A total of 503 fungal ASVs present in > 5% of chickens with NE scores of 0, 1, 2, 5, and 6 were subjected to downstream analyses. Compared with healthy mock-infected (score-0) chickens harboring a median of 162 fungal ASVs, the fungal ASVs were gradually declined in NE chickens (*P* = 0.062) (Fig. 2A), indicating reduced richness of the fungal community in NE. Although Pielou’s evenness index was similar between healthy and all groups of NE chickens (Fig. 2B), the overall alpha diversity of ileal mycobiota measured by Shannon index was not changed obviously in NE chickens in comparison with healthy controls (Fig. 2C).

Comparisons of beta diversities of the ileal mycobiome between healthy and NE chickens revealed significant differences as indicated by unweighted UniFrac (*P* = 0.001, R^2^ = 0.152) (Fig. 2D) and weighted UniFrac (*P* = 0.045, R^2^ = 0.126) (Fig. 2E). Pairwise comparisons also revealed significant differences between score-6 chickens and other groups in unweighted UniFrac as well as significantly different weighted UniFrac distances between score-0 and score-6 chickens as well as between score-0 and score-2 chickens (*P* < 0.05) (Table S1). These results suggested that severe NE induced a pronounced shift of the ileal mycobiota.

### Shifts in the mycobiota composition in the ileum of NE chickens

**Fig. 3:**
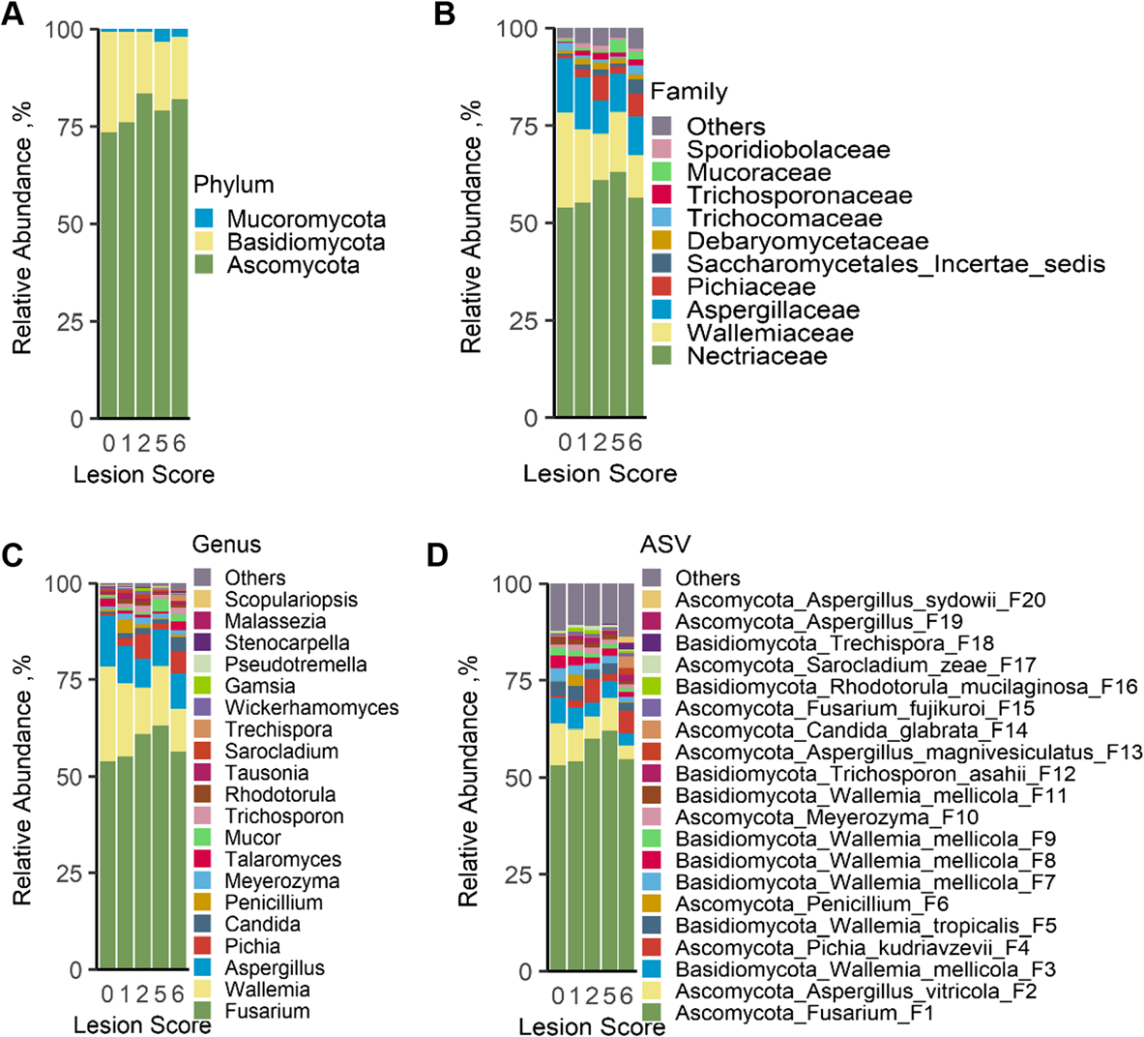
The compositions of the ileal mycobiota among chickens with different severities of NE. Chickens were sequentially infected with *E. maxima* and *C. perfringens* to induce NE and separated into five groups based on their intestinal lesion scores. Relative abundances of all phyla (**A**), top 10 families (**B**), top 20 genera (**C**), and top 20 amplicon sequence variants (ASVs) (**D**) of the ileal mycobiota were displayed

To further examine the ileal mycobiota compositional changes in response to NE, all fungi present in > 5% of the chickens were classified at phylum, family, genus, and ASV levels and compared among groups. The ileal mycobiota consisted of three phyla, 57 families, 90 genera, and 503 ASVs. The identities of top 30 fungal ASVs, averaging 92.3% of the total fungal population in all samples, were further confirmed by BLAST search of the NCBI nucleotide database (Table S2). Three phyla included Ascomycota, Basidiomycota, and Mucoromycota (Fig. 3A), with the predominant phylum Ascomycota comprising 73.5%–83.3% of the total fungal population, while the second most abundant phylum Basidiomycota constituting 15.8%–25.8% of the mycobiota (Table S3). However, none of the phyla showed a significant difference among different groups (Table S3). *Wallemia* was the only genus in the Wallemiaceae family and also the second most abundant genus in the ileum of chickens (Fig. 3B and C). *Wallemia* showed a gradual decline from 24.5% in healthy chickens to 10.1% in score-6 chickens (*P* = 0.02 and FDR = 0.08) (Table S3). By contrast, several other genera such as *Pichia, Candida*, *Trechispora*, *Pseudotremella*, and *Malassezia* were enriched in NE chickens in comparison with healthy controls (Fig. 3B, C, and Table S3).

At the ASV level, a differential response of the ileal mycobiome to NE was observed. The most abundant ASV, an unidentified *Fusarium* species (F1) comprising 53.1%–62.2% of the total fungal population showed no obvious changes among healthy and infected chickens (Fig. 3D and Table S3). However, the second most abundant ASV, *Aspergillus vitricola* (F2), accounted for 10.7% of the mycobiota in healthy broilers and was gradually reduced to 3.5% in score-6 chickens (Fig. 3D and Table S3). *Wallemia mellicola* (F3, F7, F8, F9, and F11) and *W. tropicalis* (F5) also showed a progressive decline when NE was aggravated (Table S3). Conversely, *Pichia kudriavzevii* (F4) and *Trichosporon asahii* (F12) were more abundant in chickens with NE (Table S3).

### Differential enrichment of the mycobiota in response to NE

**Fig. 4:**
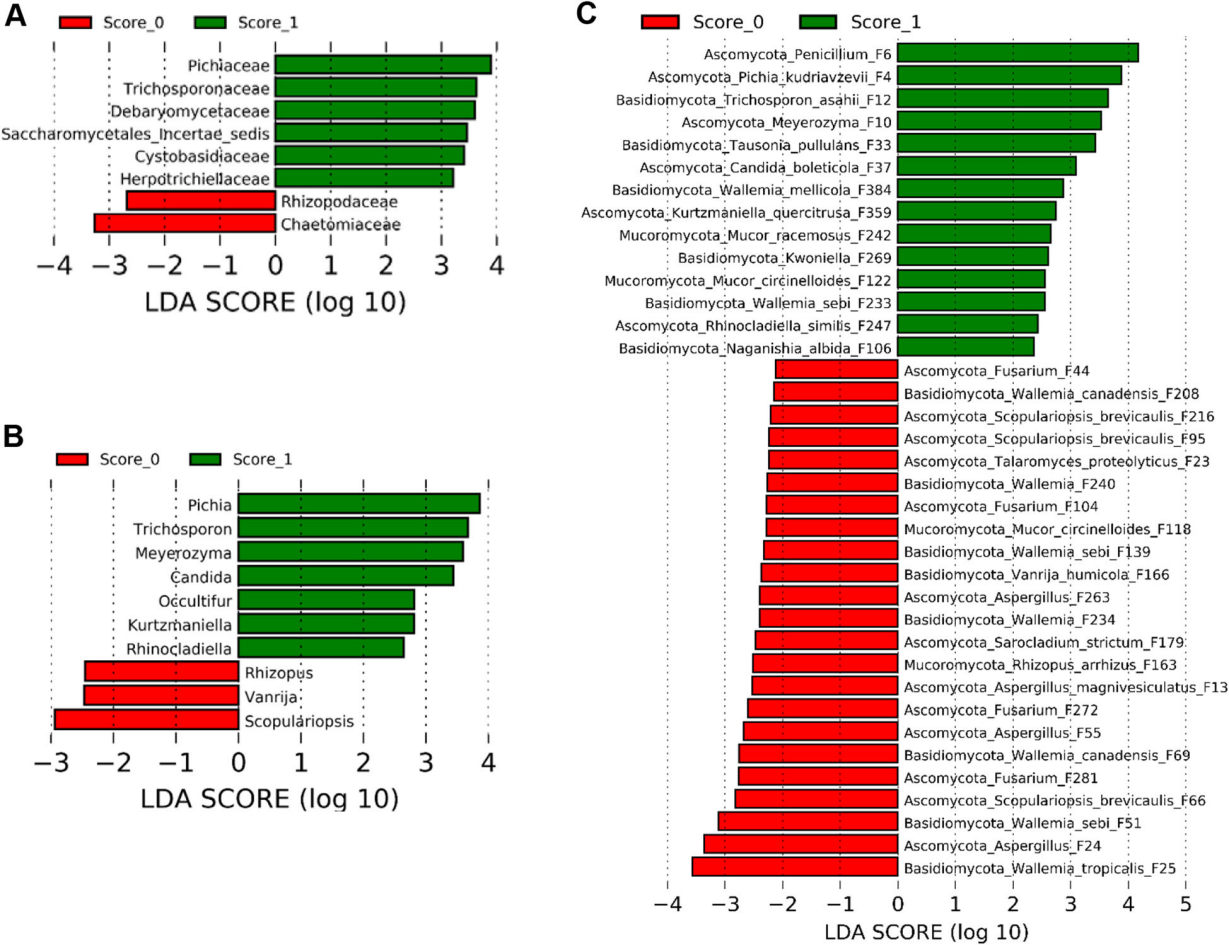
Differential enrichment of the ileal fungi between healthy and mildly infected chickens. Chickens were sequentially infected with *E. maxima* and *C. perfringens* to induce NE. Intestinal lesions in individual chickens were scored. LEfSe analysis was performed between healthy mock-infected control (score-0) and mildly infected (score-1) chickens to determine differential enrichment of fungi at the family (**A**), genus (**B**), and amplicon sequence variant (ASV) levels (**C**). The cut-off thresholds were set at *P* < 0.05 and LDA score > 2.0

To identify discriminative fungi between healthy and NE chickens, LEfSe was first performed between score-0 and score-1 chickens at the family, genus, and ASV levels among those common taxa that were present in > 20% of chickens using thresholds of *P* < 0.05 and LDA score > 2. In comparison with healthy chickens, families such as Chaetomiaceae and Rhizopodaceae were reduced, while Pichiaceae, Trichosporonaceae, Debaryomycetaceae, Saccharomycetales incertae sedis, Cystobasidiaceae, and Herpotrichiellaceae were enriched in score-1 broilers (Fig. 4A). At the genus level, mild NE diminished *Scopulariopsis*, *Vanrija*, and *Rhizopus,* but enriched *Pichia*, *Trichosporon*, *Meyerozyma*, *Candida*, *Occultifur*, *Kurtzmaniella*, and *Rhinocladiella* (Fig. 4B). A total of 37 ASVs showed differential enrichment between healthy and mild NE chickens. Multiple members of *Wallemia* (F25, F51, F69, F139, F208, F234, and F240), *Aspergillus* (F13, F24, F55, and F263), and *Fusarium* (F44, F104, and F281) as well as *Scopulariopsis brevicaulis* (F95 and F216) were reduced in mild NE, while a diverse array of fungi such as a *Penicillium* species (F6)*. P. kudriavzevii* (F4), a *Meyerozyma* species (F10), and *T. asahii* (F12) as well as two relatively rare *Wallemia* members (F233 and F384) were increased in mildly infected NE chickens (Fig. 4C). Fungi that were enriched in mild NE mainly belonged to Saccharomycetes, Tremellomycetes, and Chaetothyriales (data not shown).

**Fig. 5:**
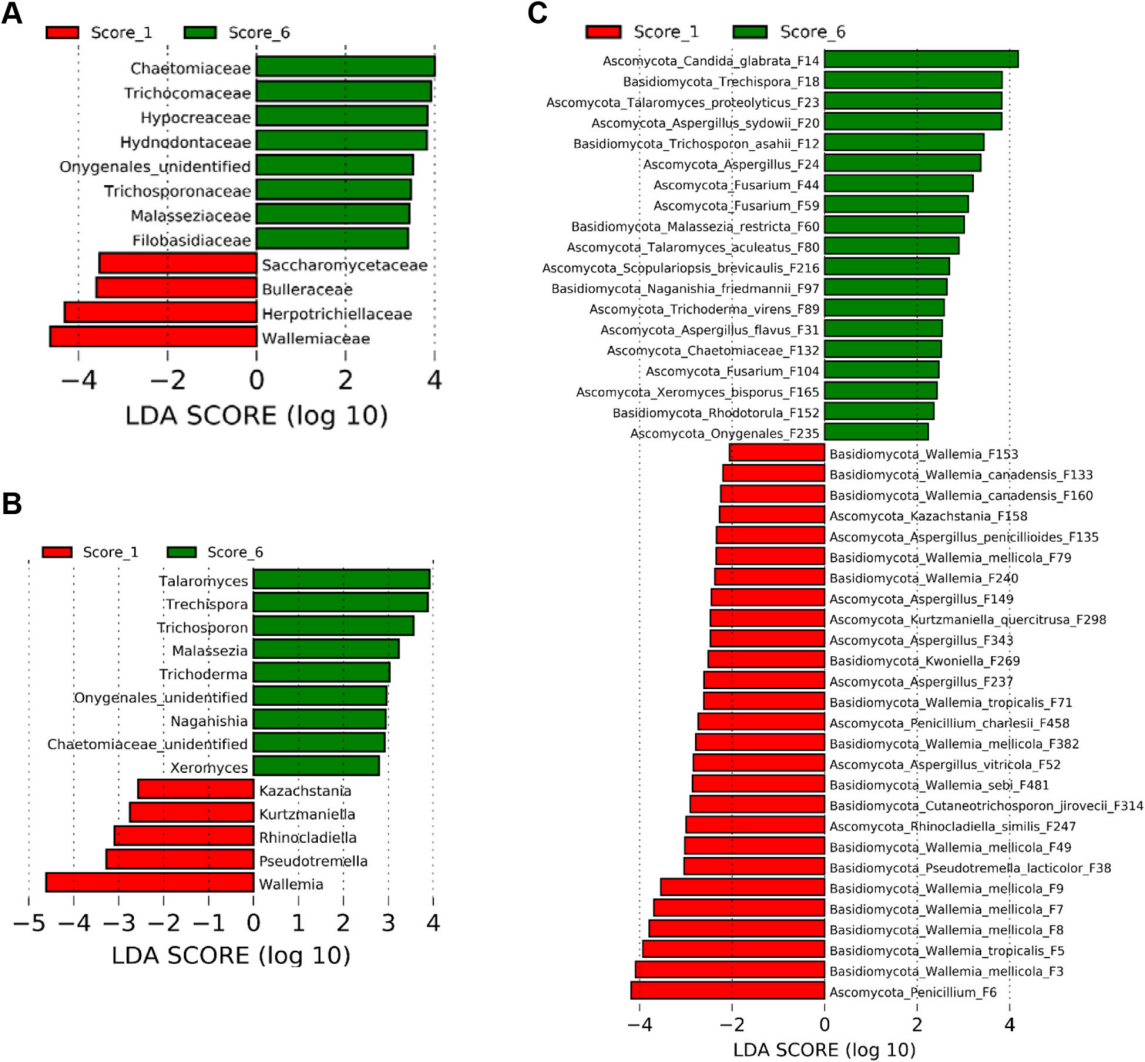
Differential enrichment of the ileal fungal taxa between chickens with mild and severe NE. Chickens were sequentially infected with *E. maxima* and *C. perfringens* to induce NE. Intestinal lesions in individual chickens were scored. LEfSe analysis was performed between chickens with mild (score-1) and severe NE (score-6) to determine differential enrichment of fungi at the family (**A**), genus (**B**), and amplicon sequence variant (ASV) levels (**C**). The cut-off thresholds were set at *P* < 0.05 and LDA score > 2.0

To further determine differentially abundant fungi in the ileum between chickens with mild and severe NE, LEfSe was performed between score-1 and score-6 chickens. At the family level, Wallemiaceae, Herpotrichiellaceae, Bulleraceae, and Saccharomycetaceae were enriched in chickens with mild NE, while Chaetomiaceae Trichocomaceae, Hypocreaceae, Hydnodontaceae, Trichosporonaceae, Malasseziaceae, Filobasidiaceae, and an unidentified family within Onygenales were more abundant in severely infected chickens (Fig. 5A). At the genus level, score-1 chickens showed an enrichment of *Wallemia*, *Pseudotremella*, *Rhinocladiella, Kurtzmaniella*, and *Kazachstania*, but genera such as *Talaromyces*, *Trechispora*, *Trichosporon*, *Malassezia*, *Trichoderma*, *Naganishia*, and *Xeromyces* were increased in score-6 chickens (Fig. 5B). At the ASV level, a total of 46 ASVs were differentially enriched between mild and severe NE. For example, *Penicillium* F6 and multiple *Wallemia* members such as *W. mellicola* (F3, F7, F8, F9, F49, F79, and F382), *W. tropicalis* (F5 and F71), *W. canadensis* (F133 and F160), and *W. sebi* (F481) were abundantly present in score-1 chickens, while enrichments of *Candida glabrata*, *T. asahii*, *Aspergillus sydowii*, and *Aspergillus flavus* were observed in score-6 chickens (Fig. 5C).

### Correlation between the ileal fungal abundance and NE severity

**Fig. 6:**
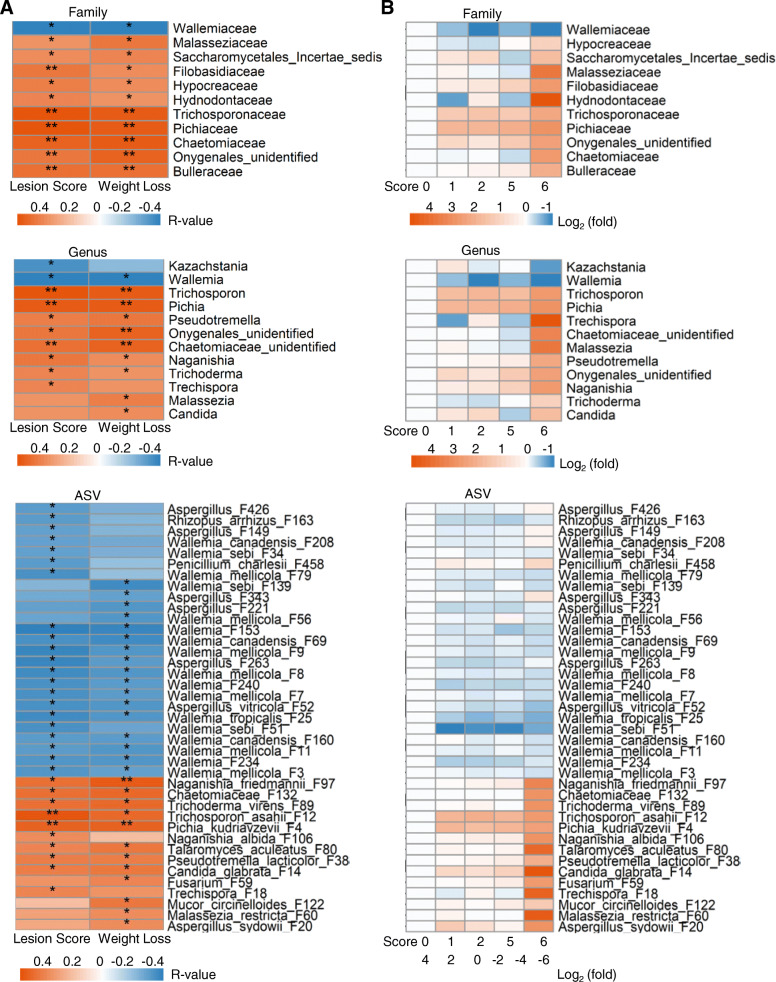
Correlation between the ileal mycobiota composition and NE severity. Chickens were sequentially infected with *E. maxima* and *C. perfringens* to induce NE. Intestinal lesions in individual chickens were scored. Spearman correlation analysis was performed between the ileal mycobiota profile and the intestinal lesion score and weight loss. **A** Spearman correlation at family, genus, and amplicon sequence variant (ASV) levels. **B** Heatmap displaying log2 transformations of fold changes in fungal abundance in NE chickens at the family, genus, and ASV levels, relative to mock-infected controls. Significance of Spearman correlations was subjected to FDR correction using the Benjamini-Hochberg procedure. *FDR < 0.05, **FDR < 0.01

To identify the ileal fungi that are strongly correlated with NE severity, Spearman’s rank correlation was performed separately between the fungal taxa present in > 20% of the chickens and two NE severity parameters (intestinal lesion score and weight loss). Among 33 families and 47 genera that were common in > 20% of samples, 11 families and 12 genera showed a significant correlation (FDR < 0.05) with at least one indicator of NE severity (Fig. 6A). While *Wallemiaceae*/*Wallemia* was negatively correlated with disease severity (FDR < 0.05), *Trichosporonaceae*/*Trichosporon, Pichiaceae*/*Pichia*, *Filobasidiaceae*/*Naganishia,* an unidentified member of Onygenales, and *Bulleraceae*/*Pseudotremella* showed a significant positive correlation (FDR < 0.05) with both NE severity indicators. Out of 198 fungal ASVs present in > 20% of chickens, 39 ASVs showed a significant correlation with NE severity, with 14 showing a positive correlation and 25 showing a negative correlation (FDR < 0.05) (Fig. 6A). For example, 17 *Wallemia* species such as *W. mellicola* (F3, F7, F8, F9, and F11), *W. tropicalis* (F25), *W. sebi* (F34 and F51) and six *Aspergillus* members (e.g., *A. vitricola* F52 and unclassified *Aspergillus*) exhibited a strong negative correlation with at least one indicator of NE severity (Fig. 6A). As revealed in a heatmap, multiple *Wallemia* members were diminished, while a diverse group of fungi were enriched in exacerbated NE (Fig. 6B).

**Fig. 7:**
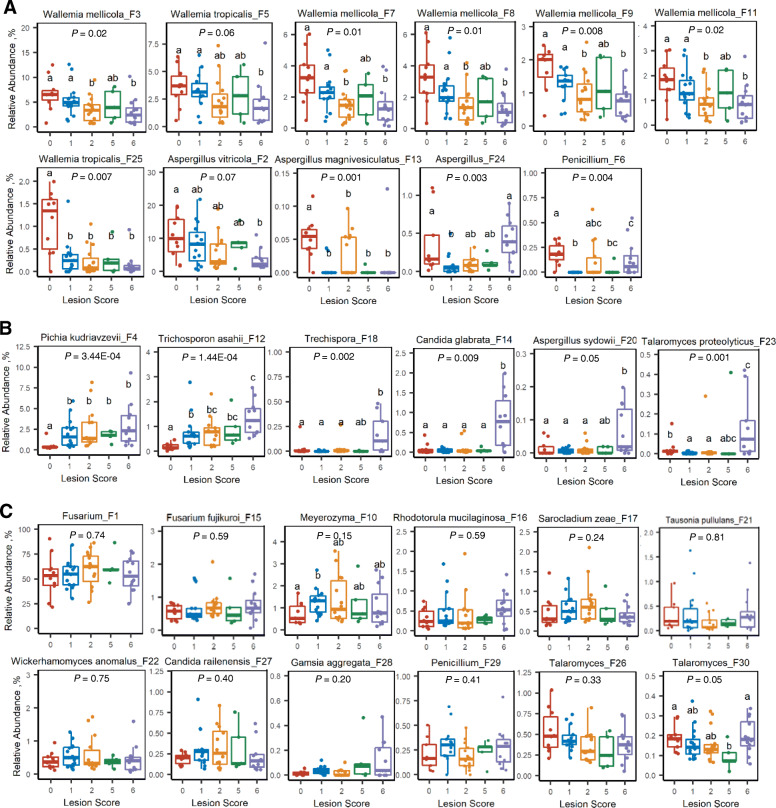
Relative abundances of top 30 ileal fungal amplicon sequence variants (ASVs) among chickens with different severities of NE. Ileal fungi were diminished (**A**), increased (**B**), or unaltered (**C**) in NE. Each box indicated median, 25th and 75th percentiles, while whiskers of the box plots extended to 1.5 interquartile range. Significance was determined using Kruskal-Wallis test and was indicated on the top of each plot. Groups not sharing common superscripts were significantly different at *P* < 0.05 as measured by pair-wise Mann–Whitney U test

A closer examination of the 30 most abundant fungal ASVs further revealed different patterns of the fungal change in response to NE. While *W. mellicola* (F3, F7, F8, F9, and F11) and *W. tropicalis* (F5) were gradually diminished in more severe NE; *W. tropicalis* F25 showed an abrupt decrease even in chickens with mild NE (Fig. 7A), suggesting both inter-species and intra-species variations of *Wallemia* in response to NE. Similarly, different sensitivities to NE were observed among *Aspergillus* species. While *A. vitricola* (F2) displayed a progressive decline, *A. magnivesiculatus* (F13) was abruptly abolished in all NE chickens (Fig. 7A). On the other hand, *A. sydowii* (F20) appeared to be enriched only in chickens with severe NE (Fig. 7B). An unspecified *Trechispora* (F18), *C. glabrata* (F14), and *Talaromyces proteolyticus* (F23) were also sharply increased in severe NE chickens (*P* < 0.05), but *P. kudriavzevii* (F4) and *T. asahii* (F12) showed a progressive increase with NE severity (Fig. 7B). Notably, nearly 50% of top 30 fungal ASVs showed no obvious shift change in response to NE including the most abundant *Fusarium* ASV (F1) (Fig. 7C). Perhaps as confirmation, another *Fusarium* (*F. fujikuroi* F15) was unaltered in NE either (Fig. 7C).

### Correlation between the ileal mycobiota and microbiota in the context of NE

**Fig. 8:**
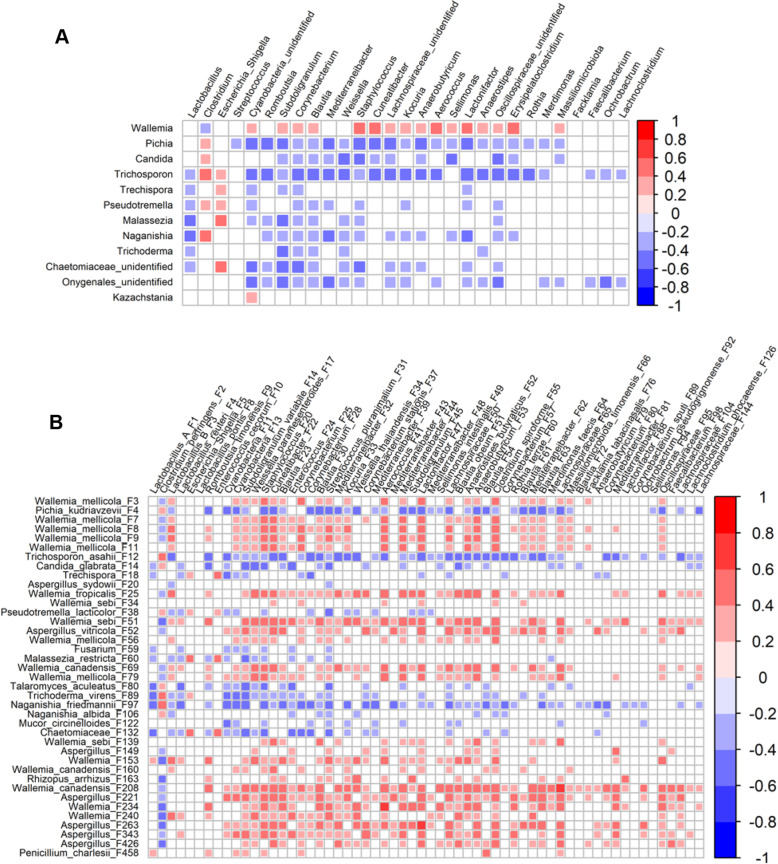
The ileal fungal-bacterial correlations in chickens with NE. Spearman correlation was performed to evaluate potential interactions between the ileal fungi and bacteria at the genus (**A**) and amplicon sequence variant (ASV) levels (**B**). Only fungal and bacterial taxa that were present in > 20% of chickens and also correlated significantly with NE severity were included in the analysis. Significant positive and negative correlations (FDR < 0.05) were denoted by red and blue squares, respectively, while the strength of a correlation was indicated by the extent of the color. White squares indicated nonsignificant correlations

In addition to the mycobiota, the bacterial microbiota of the ileum was also investigated by 16S rRNA gene sequencing. Spearman correlation analysis was conducted between the ileal fungi and bacteria to explore their potential interactions in the context of NE. Among 12 fungal and 29 bacterial genera that were commonly present in > 20% of the chickens and also showed a significant correlation with NE severity (FDR < 0.05), *Wallemia* showed a negative correlation with *Clostridium,* but a positive correlation with Cyanobacteria, *Subdoligranulum*, *Corynebacterium*, *Blautia*, *Staphylococcus*, *Cuneatibacter*, *Aerococcus*, *Lactonifactor*, *Oscillospiraceae*, *Erysipelatoclostridium,* and an unidentified genus in *Lachnospiraceae* (Fig. 8A). Positive correlations also occurred between *Clostridium* and fungal genera including *Pichia*, *Candida*, *Trichosporon*, *Pseudotremella*, and *Naganishia*, while a majority of the fungal-bacterial interactions were negative (Fig. 8A).

At the ASV level, among 39 fungal and 64 bacterial ASVs showing a significant correlation with the severity of NE (FDR < 0.05), positive correlations were common among NE-diminished fungi such as *Wallemia* species and NE-reduced bacteria (majorly lactic acid bacteria and SCFA-producing bacteria, such as group B *Lactobacillus*, *L. reuteri*, *Subdoligranulum variabile*, *Weissella* species, and *Blautia* species) (Fig. 8B). On the contrary, negative interactions mainly occurred between NE-enriched fungi (e.g., *P. kudriavzevii* F4, *T. asahii* F12, *C. glabrata* F14, and *Naganishia friedmannii* F97) and bacterial species that were decreased in NE (Fig. 8B).

## Discussion

The bacterial microbiota and fungal mycobiota residing in the GI tract contribute to the health or diseases of the host [1–5]. Disturbance in the intestinal microbiota has been linked to chicken NE [17], while the involvement of the mycobiome in NE is yet to be investigated. In the current study, we unraveled an altered ileal fungal community in NE-afflicted chickens using ITS2 amplicon sequencing and further identified a number of fungi that are strongly correlated with the severity of NE. Our study also revealed the mycobiota-microbiota correlations in NE, suggesting that the mycobiome, in addition to the microbiome, might be potentially involved in NE and targeted to mitigate NE, although direct experimental evidence is needed.

We found that chicken intestinal mycobiota is dominated by Ascomycota followed by Basidiomycota. The two fungal phyla make up 97%–99% of the ileal mycobiota in broilers, in agreement with a recent report [14]. Similarly, Ascomycota and Basidiomycota are two major fungal phyla found in the human GI tract [8, 9]. Ascomycota is also the most abundant fungal phylum in the intestine of piglets [34]. In the present study, *Fusarium* is the most predominant in the chicken ileum, regardless of the health status. *Fusarium*, *Wallemia*, and *Aspergillus* collectively comprise 84% of the ileal fungal community, while *Pichia*, *Candida*, *Penicillium*, *Mucor*, and *Trichosporon* also show a high prevalence in the chicken ileum. Similar to our results, *Candida*, *Trichosporon*, and *Rhodotorula* are major fungi isolated from chicken feces [35], which are also among predominant fungi in the GI tract of healthy turkeys [36]. As for the source of the intestinal fungi, *Fusarium, Aspergillus*, *Penicillium*, and *Mucor* were found to be prevalent in corn, soybean meal, and finished poultry feed [37, 38], suggesting that the intestinal mycobiota is mainly originated from the feed.

In this study, we found that richness, but not evenness or Shannon index, of the ileal mycobiota tends to decrease in NE, which is similar to earlier studies with IBD and ulcerative colitis patients [3, 8], although other studies described no obvious changes in fungal richness between Crohn’s disease patients and healthy cohorts [13, 39]. One major finding of our research is that the total ileal fungal population is drastically reduced in severe NE. The fungal population approximately constitutes approximately 0.1% of the total bacteria in the ileum of healthy chickens, which is consistent with the previous report in humans and mice [40], but is gradually diminished in NE, with an approximately 50-fold reduction in severely infected, score-6 chickens. On the other hand, the total bacterial load is increased by 2- to 3-fold in NE chickens. As a result, a progressive decline in the fungal-bacterial ratio occurs in exacerbated NE. Interestingly, the fungal load and the fungal-bacterial ratio are increased in patients with Crohn’s disease [39]. More research is warranted to further investigate whether colonization and proliferation of *C. perfringens* in the small intestine in NE lead to diminished mycobiota.

In this study, we observed an obvious difference in the sensitivity to NE among the ileal fungal taxa. Among the 30 most abundant fungi, approximately 40% remain unchanged, while another 40% are declined and the remaining 20% are enriched in NE. Among those that are altered in NE, some show a gradual increase or decrease, while others are changed abruptly. For example, *C. glabrata* (F14), *A. sydowii* (F20), *T. proteolyticus* (F23) and a *Trechispora* species (F18) are enriched only in the ileum of severally infected chickens.

Among those fungi that are dramatically reduced in NE are a number of *Wallemia* taxa that are commonly found in the air, house dust, soils, and plants [11, 41]. *Wallemia* is also a commensal in the intestine of humans and mice [11]. In this study, we revealed that several *Wallemia* species such as *W. mellicola*, *W. tropicalis*, *W. sebi*, and *W. canadensis* are negatively correlated with NE severity. Consistently, *Wallemia* is reported to produce UCA1064-A and 1064-B with beneficial antitumor, antifungal, and antimicrobial activities [42, 43]. However, *W. sebi* and *W. mellicola* have been found to be associated with skin infections and allergic airway diseases [10, 11]. Further studies are needed to understand the involvement of *Wallemia* in NE.

Apart from *Wallemia*, *Aspergillus* is also apparently reduced in NE. *A. vitricola*, *A. magnivesiculatus*, and several unidentified *Aspergillus* species are negatively correlated with NE severity. *Aspergillus* is ubiquitous in feed and can cause aspergillosis in avian species or humans [44]. Although it can be a source of dietary mycotoxins with a detrimental effect on poultry health and performance [37], *Aspergillus* also produces beneficial metabolites such as lovastatin, terreulactones, 11-α hydroprogesterone, quadrone, and terpeptin [45]. The potential of *Aspergillus* for disease resistance in poultry warrants further investigation.

Along with a striking decrease in *Wallemia* and *Aspergillus*, other fungi such as *P. kudriavzevii* (teleomorph of *Candida krusei*), *C. glabrata*, *M. restricta*, *T. asahii*, and *N. friedmannii* are enriched in the ileum of chickens with NE, especially in severe NE. *P. kudriavzevii* isolated from chicken feces exhibits probiotic properties in vitro [46]. With a capacity to bind to aflatoxin B1, dietary supplementation of *P. kudriavzevii* mitigates the adverse effect of aflatoxin B1 on the growth performance of broilers [47]. *Candida* species are a part of commensal mycobiota in the GI tract but may cause candidiasis in poultry and humans [36, 41, 44]. *Candida* infections most frequently occur in the upper GI tract of chickens and may result in growth retardation or even mortality [44]. We revealed a positive correlation between *C. glabrata* and NE severity, which is in agreement with reports that *Candida* such as *C. albicans*, *C. glabrata*, and *C. tropicalis* are associated with IBD [8, 13, 39]. The role of *P. kudriavzevii* and *Candida* overgrowth in NE is not clear, and further studies are warranted.

*Malassezia* is a commensal fungus that colonizes not only the skin but also in the GI tract of humans and animals [48]. *M. restricta* has been found to be associated with IBD and several other intestinal inflammatory disorders in humans [48]. *M. restricta*, with potent pro-inflammatory properties, is enriched in Crohn’s disease patients, and oral administration of *M. restricta* exacerbates colitis in mice [9]. *T. asahii* is another commensal fungus that can cause opportunistic infections [14, 49]. In agreement with expanded intestinal *Trichosporon* in IBD patients [8], we found that *T. asahii* is positively correlated with NE severity in chickens. In addition, *N. friedmannii* (formerly *Cryptococcus friedmannii)* is reported to cause onychomycosis in humans [50]. The overgrowth of opportunistic fungi in NE-infected chickens might increase susceptibility of chickens to mycosis and probably threat public health.

Fungi and bacteria co-colonize the GI tract and interact with each other directly or indirectly through physical contact, microbial metabolites, and modification of immune status [4, 51]. The cross-talk between the mycobiota and the microbiota is critical for maintaining intestinal homeostasis [4, 51] and has been demonstrated in turkeys [15] and human IBD [8, 13]. We revealed a dramatic shift of the ileal microbiota in NE (unpublished). The current study has further revealed a strong positive or negative correlation between a number of fungal and bacterial taxa in NE. For example, most *Wallemia* species are correlated negatively with *C. perfringens* colonization in the ileum but correlated positively with a number of SCFA-producing bacteria, consistent with an earlier report on a positive association between *Wallemia* and SCFA-producing *Oscillospiraceae* [52]. On the other hand, *P. kudriavzevii* (F4), *T. asahii* (F12)*, C. glabrata* (F14)*,* and *M. restricta* (F60) are positively correlated with *C. perfringens* with a negative correlation with SCFA-producing bacteria and often lactic acid bacteria. Such an antagonism between *C. glabrata* and lactic acid bacteria (e.g., *Lactobacillus* and *Weissella*) was also reported earlier [53, 54]. It will be important to study the role of the fungi-bacteria interplay in the development of NE and whether such interactions can be explored for control and prevention of NE.

*Eimeria* infection is an important predisposing factor for NE in poultry with the ability to cause damage to the intestinal epithelium, providing niches or nutrients to facilitate the colonization and proliferation of *C. perfringens* [18]. *Eimeria* in conjunction with *C. perfringens* challenge is thus the most commonly used approach to experimentally induce NE, while the same dose of *Eimeria* or *C. perfringens* alone causes no or only mild intestinal lesions [18]. Consistently, co-infection with *Eimeria* and *C. perfringens* causes more pronounced microbiota changes than inoculation separately with *Eimeria* or *C. perfringens* [55, 56]. However, the impact of *Eimeria* or *C. perfringens* on the intestinal mycobiota is currently unknown and warrants further investigation.

## Conclusion

This study revealed for the first time dysbiosis of the chicken ileal mycobiota induced by NE. The total fungal population is drastically reduced in NE and alterations in the mycobiota are more pronounced in exacerbated NE. Furthermore, we reported positive and negative correlations between a number of fungi and bacteria. These findings suggest a possible role of the intestinal mycobiota in NE pathogenesis and highlight the mycobiota as a new potential target for NE management in poultry.

## Supplementary Information

**Additional file 1: Table S1.** Pairwise comparison of beta diversity of the ileal mycobiota between healthy and NE chickens. **Table S2.** Taxonomy of top 30 fungal amplicon sequence variants (ASVs) in the chicken ileum identified through a BLAST search of the NCBI nucleotide database. **Table S3.** Relative abundance (%) of top fungal taxa in the chicken ileum.

## Data Availability

The raw sequencing reads of this study have been deposited in the NCBI Sequence Read Archive (SRA) database under BioProject PRJNA725022.

## Abbreviations

ANOVA: Analysis of variance
ASV: Amplicon sequence variant
BW: Body weight
CFU: Colony-forming unit
FDR: False discovery rate
GI: Gastrointestinal
IBD: Inflammatory bowel disease
ITS2: Internal transcribed spacer 2
LDA: Linear discriminate analysis
LEfSe: Linear discriminant analysis effect size
NE: Necrotic enteritis
NRC: National Research Council
OTU: Operational taxonomic unit
PCoA: Principal coordinates analysis
PERMANOVA: Permutational multivariate analysis of variance
qPCR: Quantitative PCR
SCFA: Short-chain fatty acid

## References

[1] Peixoto RS, Harkins DM, Nelson KE (2021). Advances in microbiome research for animal health. Annu Rev Anim Biosci.

[2] Fan Y, Pedersen O (2021). Gut microbiota in human metabolic health and disease. Nat Rev Microbiol.

[3] Li XV, Leonardi I, Iliev ID (2019). Gut mycobiota in immunity and inflammatory disease. Immunity..

[4] Santus W, Devlin JR, Behnsen J (2021). Crossing kingdoms: how the mycobiota and fungal-bacterial interactions impact host health and disease. Infect Immun.

[5] Wu X, Xia Y, He F, Zhu C, Ren W (2021). Intestinal mycobiota in health and diseases: from a disrupted equilibrium to clinical opportunities. Microbiome..

[6] Tso GHW, Reales-Calderon JA, Tan ASM, Sem X, Le GTT, Tan TG (2018). Experimental evolution of a fungal pathogen into a gut symbiont. Science..

[7] Shao TY, Ang WXG, Jiang TT, Huang FS, Andersen H, Kinder JM (2019). Commensal *Candida albicans* positively calibrates systemic Th17 immunological responses. Cell Host Microbe.

[8] Sokol H, Leducq V, Aschard H, Pham HP, Jegou S, Landman C, Cohen D, Liguori G, Bourrier A, Nion-Larmurier I, Cosnes J, Seksik P, Langella P, Skurnik D, Richard ML, Beaugerie L (2017). Fungal microbiota dysbiosis in IBD. Gut..

[9] Limon JJ, Tang J, Li D, Wolf AJ, Michelsen KS, Funari V (2019). Malassezia is associated with Crohn‘s disease and exacerbates colitis in mouse models. Cell Host Microbe.

[10] Skalski JH, Limon JJ, Sharma P, Gargus MD, Nguyen C, Tang J, Coelho AL, Hogaboam CM, Crother TR, Underhill DM (2018). Expansion of commensal fungus Wallemia mellicola in the gastrointestinal mycobiota enhances the severity of allergic airway disease in mice. PLoS Pathog.

[11] Zajc J, Gunde-Cimerman N (2018). The genus Wallemia-from contamination of food to health threat. Microorganisms..

[12] Coker OO, Nakatsu G, Dai RZ, Wu WKK, Wong SH, Ng SC, Chan FKL, Sung JJY, Yu J (2019). Enteric fungal microbiota dysbiosis and ecological alterations in colorectal cancer. Gut..

[13] Hoarau G, Mukherjee PK, Gower-Rousseau C, Hager C, Chandra J, Retuerto MA (2016). Bacteriome and mycobiome interactions underscore microbial dysbiosis in familial Crohn‘s disease. mBio.

[14] Robinson K, Xiao Y, Johnson TJ, Chen B, Yang Q, Lyu W (2020). Chicken intestinal mycobiome: initial characterization and its response to bacitracin methylene disalicylate. Appl Environ Microbiol.

[15] Ward TL, Weber BP, Mendoza KM, Danzeisen JL, Llop K, Lang K (2019). Antibiotics and host-tailored probiotics similarly modulate effects on the developing avian microbiome, mycobiome, and host gene expression. mBio.

[16] Wade B, Keyburn A (2015). The true cost of necrotic enteritis. World Poult.

[17] Antonissen G, Eeckhaut V, Van Driessche K, Onrust L, Haesebrouck F, Ducatelle R (2016). Microbial shifts associated with necrotic enteritis. Avian Pathol.

[18] Shojadoost B, Vince AR, Prescott JF (2012). The successful experimental induction of necrotic enteritis in chickens by Clostridium perfringens: a critical review. Vet Res.

[19] Cooper KK, Songer JG (2010). Virulence of Clostridium perfringens in an experimental model of poultry necrotic enteritis. Vet Microbiol.

[20] Al-Badri R, Barta JR (2012). The kinetics of oocyst shedding and sporulation in two immunologically distinct strains of Eimeria maxima, GS and M6. Parasitol Res.

[21] Latorre JD, Adhikari B, Park SH, Teague KD, Graham LE, Mahaffey BD, Baxter MFA, Hernandez-Velasco X, Kwon YM, Ricke SC, Bielke LR, Hargis BM, Tellez G (2018). Evaluation of the epithelial barrier function and ileal microbiome in an established necrotic enteritis challenge model in broiler chickens. Front Vet Sci.

[22] diCenzo GC, Finan TM (2017). The divided bacterial genome: structure, function, and evolution. Microbiol Mol Biol Rev.

[23] De Fine Licht HH, Hajek AE, Eilenberg J, Jensen AB (2016). Utilizing genomics to study entomopathogenicity in the fungal phylum entomophthoromycota: a review of current genetic resources. Adv Genet.

[24] White TJ, Bruns T, Lee S, Taylor J, Innis MA, Gelfand DH, Sninsky JJ, White TJ (1990). Amplification and direct sequencing of fungal ribosomal RNA genes for phylogenetics. PCR protocols: a guide to methods and applications.

[25] Bolyen E, Rideout JR, Dillon MR, Bokulich NA, Abnet CC, Al-Ghalith GA (2019). Reproducible, interactive, scalable and extensible microbiome data science using QIIME 2. Nat Biotechnol.

[26] Amir A, McDonald D, Navas-Molina JA, Kopylova E, Morton JT, Zech Xu Z (2017). Deblur rapidly resolves single-nucleotide community sequence patterns. mSystems.

[27] Paulson JN, Stine OC, Bravo HC, Pop M (2013). Differential abundance analysis for microbial marker-gene surveys. Nat Methods.

[28] McMurdie PJ, Holmes S (2013). phyloseq: an R package for reproducible interactive analysis and graphics of microbiome census data. PLoS One.

[29] Lozupone C, Knight R (2005). UniFrac: a new phylogenetic method for comparing microbial communities. Appl Environ Microbiol.

[30] Segata N, Izard J, Waldron L, Gevers D, Miropolsky L, Garrett WS, Huttenhower C (2011). Metagenomic biomarker discovery and explanation. Genome Biol.

[31] Wickham H (2016). ggplot2: elegant graphics for data analysis. 2nd ed.

[32] Wang X, Tsai T, Deng F, Wei X, Chai J, Knapp J, Apple J, Maxwell CV, Lee JA, Li Y, Zhao J (2019). Longitudinal investigation of the swine gut microbiome from birth to market reveals stage and growth performance associated bacteria. Microbiome..

[33] Liu J, Stewart SN, Robinson K, Yang Q, Lyu W, Whitmore MA, Zhang G (2021). Linkage between the intestinal microbiota and residual feed intake in broiler chickens. J Anim Sci Biotechnol.

[34] Hu J, Nie Y, Chen J, Zhang Y, Wang Z, Fan Q (2016). Gradual changes of gut microbiota in weaned miniature piglets. Front Microbiol.

[35] Subramanya SH, Sharan NK, Baral BP, Hamal D, Nayak N, Prakash PY, Sathian B, Bairy I, Gokhale S (2017). Diversity, in-vitro virulence traits and antifungal susceptibility pattern of gastrointestinal yeast flora of healthy poultry, Gallus gallus domesticus. BMC Microbiol.

[36] Sokol I, Gawel A, Bobrek K (2018). The prevalence of yeast and characteristics of the isolates from the digestive tract of clinically healthy turkeys. Avian Dis.

[37] Ghaemmaghami SS, Modirsaneii M, Khosravi AR, Razzaghi-Abyaneh M (2016). Study on mycoflora of poultry feed ingredients and finished feed in Iran. Iran J Microbiol.

[38] Oliveira GR, Ribeiro JM, Fraga ME, Cavaglieri LR, Direito GM, Keller KM, Dalcero AM, Rosa CA (2006). Mycobiota in poultry feeds and natural occurrence of aflatoxins, fumonisins and zearalenone in the Rio de Janeiro state, Brazil. Mycopathologia.

[39] Liguori G, Lamas B, Richard ML, Brandi G, da Costa G, Hoffmann TW, di Simone MP, Calabrese C, Poggioli G, Langella P, Campieri M, Sokol H (2016). Fungal dysbiosis in mucosa-associated microbiota of Crohn‘s disease patients. J Crohns Colitis.

[40] Li J, Chen D, Yu B, He J, Zheng P, Mao X, Yu J, Luo J, Tian G, Huang Z, Luo Y (2018). Fungi in gastrointestinal tracts of human and mice: from community to functions. Microb Ecol.

[41] Limon JJ, Skalski JH, Underhill DM (2017). Commensal fungi in health and disease. Cell Host Microbe.

[42] Jančič S, Frisvad JC, Kocev D, Gostinčar C, Džeroski S, Gunde-Cimerman N (2016). Production of secondary metabolites in extreme environments: food- and airborne Wallemia spp. produce toxic metabolites at hypersaline conditions. PLoS One.

[43] Takahashi I, Maruta R, Ando K, Yoshida M, Iwasaki T, Kanazawa J (1993). UCA1064-B, a new antitumor antibiotic isolated from Wallemia sebi: production, isolation and structural determination. J Antibiot (Tokyo).

[44] Dykstra MJ, Charlton BR, Chin RP, Barnes HJ, Swayne DE, Glisson JR, McDougald LR, Nolan LK, Suarez DL, Nair VL (2013). Fungal infections. Diseases of poultry.

[45] Ashtekar N, Anand G, Prakash PY, Rajeshkumar KC, Singh J, Gehlot P (2021). Aspergillus terreus: taxonomy, biology, and bioactive secondary metabolites with potential applications. New and future developments in microbial biotechnology and bioengineering.

[46] García-Hernández Y, Rodríguez Z, Brandão LR, Rosa CA, Nicoli JR, Elías Iglesias A, Peréz-Sanchez T, Salabarría RB, Halaihel N (2012). Identification and in vitro screening of avian yeasts for use as probiotic. Res Vet Sci.

[47] Magnoli AP, Rodriguez MC, Poloni VL, Rojo MC, Combina M, Chiacchiera SM, Dalcero AM, Cavaglieri LR (2016). Novel yeast isolated from broilers‘ feedstuff, gut and faeces as aflatoxin B_1_ adsorbents. J Appl Microbiol.

[48] Spatz M, Richard ML (2020). Overview of the potential role of Malassezia in gut health and disease. Front Cell Infect Microbiol.

[49] Duarte-Oliveira C, Rodrigues F, Gonçalves SM, Goldman GH, Carvalho A, Cunha C (2017). The cell biology of the Trichosporon-host interaction. Front Cell Infect Microbiol.

[50] Ekhtiari M, Farahyar S, Falahati M, Razmjou E, Ashrafi-Khozani M, Ghasemi Z, Abbasi-Nejat Z (2017). The first report of onychomycosis caused by Cryptococcus friedmannii (Naganishia friedmannii) a basidiomycetous yeast. Med Mycol Case Rep.

[51] Kruger W, Vielreicher S, Kapitan M, Jacobsen ID, Niemiec MJ (2019). Fungal-bacterial interactions in health and disease. Pathogens..

[52] Lin M, Feng L, Cheng Z, Wang K (2020). Effect of ethanol or lactic acid on short chain fatty acid production and microbial community in short-term sequentially transfers by ruminal fermented with wheat straw in vitro. Process Biochem.

[53] Boris S, Barbés C (2000). Role played by lactobacilli in controlling the population of vaginal pathogens. Microbes Infect.

[54] Fan D, Coughlin LA, Neubauer MM, Kim J, Kim MS, Zhan X, Simms-Waldrip TR, Xie Y, Hooper LV, Koh AY (2015). Activation of HIF-1α and LL-37 by commensal bacteria inhibits Candida albicans colonization. Nat Med.

[55] Stanley D, Wu SB, Rodgers N, Swick RA, Moore RJ (2014). Differential responses of cecal microbiota to fishmeal, Eimeria and Clostridium perfringens in a necrotic enteritis challenge model in chickens. PLoS One.

[56] Yang WY, Lee Y, Lu H, Chou CH, Wang C (2019). Analysis of gut microbiota and the effect of lauric acid against necrotic enteritis in Clostridium perfringens and Eimeria side-by-side challenge model. PLoS One.

