# Supplementary material for: Perturbations of the ileal mycobiota by necrotic enteritis in broiler chickens
Source: J Anim Sci Biotechnol. 2021 Oct 9;12:107. doi: 10.1186/s40104-021-00628-5 (PMC8501549; doi:10.1186/s40104-021-00628-5)
Supplement: Supplementary file 1 — Additional file 1: Table S1. Pairwise comparison of beta diversity of the ileal mycobiota between healthy and NE chickens. Table S2. Taxonomy of top 30 fungal amplicon sequence variants (ASVs) in the chicken ileum identified through a BLAST search of the NCBI nucleotide database. Table S3. Relative abundance (%) of top fungal taxa in the chicken ileum. [file 40104_2021_628_MOESM1_ESM.docx]

**Supplementary Information**

**Table S1. Pairwise comparison of beta diversity of the ileal mycobiota between healthy and NE chickens**

| Lesion Score | 0 | 1 | 2 | 5 | 6 |
| --- | --- | --- | --- | --- | --- |
| 0 |  | 0.272  (0.049) | 0.017  (0.165) | 0.235  (0.102) | 0.023  (0.161) |
| 1 | 0.010  (0.084) |  | 0.083  (0.073) | 0.520  (0.040) | 0.106  (0.076) |
| 2 | 0.151  (0.056) | 0.208  (0.043) |  | 0.588  (0.035) | 0.838  (0.016) |
| 5 | 0.220  (0.090) | 0.790  (0.037) | 0.590  (0.044) |  | 0.581  (0.044) |
| 6 | 0.001  (0.140) | 0.001  (0.170) | 0.001  (0.124) | 0.009  (0.134) |  |

**Note:** P-values and R^2^ (in parentheses) of pairwise comparisons of unweighted (shaded) and weighted UniFrac distances of the ileal mycobiota in chickens with differing severities of NE were determined by PERMANOVA.

**Table S2**. **Taxonomy of top 30 fungal amplicon sequence variants (ASVs) in the chicken ileum identified through a BLAST search of the NCBI nucleotide database**

| **ASV** | **Top Hits** | **E-value** | **Identity (%)** | **Accession no.** |
| --- | --- | --- | --- | --- |
| Fusarium_F1 | *Fusarium verticillioides*; *Fusarium subglutinans* | 2e-153 | 100.00% | MT598827.1  MT598164.1 |
| Aspergillus_vitricola_F2 | *Aspergillus vitricola* | 5e-158 | 100.00% | NR_135335.1 |
| Wallemia_mellicola_F3 | *Wallemia mellicola* | 2e-153 | 100.00% | KJ409889.1 |
| Pichia kudriavzevii_F4 | *Pichia kudriavzevii* | 5e-158 | 100.00% | NR_131315.1 |
| Wallemia_tropicalis_F5 | *Wallemia tropicalis* | 2e-153 | 100.00% | KM217176.1 |
| Penicillium_F6 | *Penicillium fimorum*; *Penicillium cyclopium*; *Penicillium griseofulvum* | 5e-158 | 100.00% | NR_153267.1  NR_163529.1  NR_103692.1 |
| Wallemia_mellicola_F7 | *Wallemia mellicola* | 2e-153 | 100.00% | KJ494619.1 |
| Wallemia_mellicola_F8 | *Wallemia mellicola* | 2e-153 | 100.00% | KJ409893.1  AY328917.1 |
| Wallemia_mellicola_F9 | *Wallemia mellicola* | 2e-153 | 100.00% | KJ494624.1 |
| Meyerozyma_F10 | *Meyerozyma caribbica*; *Meyerozyma carpophila* | 5e-158 | 100.00% | NR_149348.1  NR_152984.1 |
| Wallemia_mellicola_F11 | *Wallemia mellicola* | 2e-153 | 100.00 | KJ494626.1  KJ409881.1 |
| Trichosporon_asahii_F12 | *Trichosporon asahii* | 5e-158 | 100.00% | NR_073341.1 |
| Aspergillus_magnivesiculatus_F13 | *Aspergillus magnivesiculatus* | 1e-151 | 99.67% | KY087664.1 |
| Candida_glabrata_F14 | *Candida glabrata* | 2e-153 | 100.00% | MT300279.1  LR757916.1 |
| Fusarium_fujikuroi_F15 | *Fusarium fujikuroi* | 5e-158 | 100.00% | NR_111889.1 |
| Rhodotorula_mucilaginosa_F16 | *Rhodotorula mucilaginosa* | 5e-158 | 100.00 | NR_073296.1 |
| Sarocladium_zeae_F17 | *Sarocladium zeae* | 9e-146 | 100.00% | NR_130685.1 |
| Trechispora_F18 | *Trechispora* sp. | 3e-122 | 93.77% | KJ140534.1 |
| Aspergillus_F19 | *Aspergillus austroafricanus*; *Aspergillus tabacinus*; *Aspergillus protuberus* | 5e-158 | 100.00% | NR_135443.1  NR_135361.1  NR_135353.1 |
| Aspergillus_ sydowii_F20 | *Aspergillus sydowii* | 9e-156 | 100.00% | NR_131259.1 |
| Tausonia_pullulans_F21 | *Tausonia pullulans* | 2e-153 | 100.00% | MK782486.1  MK794149.1 |
| Wickerhamomyces_anomalus_F22 | *Wickerhamomyces anomalus* | 2e-153 | 100.00% | MT645457.1  MT645456.1 |
| Talaromyces_proteolyticus_F23 | *Talaromyces proteolyticus* | 1e-140 | 97.66% | KF196919.1 |
| Aspergillus_F24 | *Aspergillus costiformis*; *Aspergillus chevalieri* |  | 100.00% | NR_135434.1  NR_135340.1 |
| Wallemia_tropicalis_F25 | *Wallemia tropicalis* | 3e-152 | 100.00% | MK361154.1  KM217176.1 |
| Talaromyces_F26 | *Talaromyces wortmannii*; *Talaromyces variabilis* | 2e-153 | 100.00% | MT079310.1  MT072077.1 |
| Candida_railenensis_F27 | *Candida railenensis* | 5e-158 | 100.00% | NR_077080.1 |
| Gamsia_aggregata_F28 | *Gamsia aggregata* | 2e-153 | 100.00% | MH859298.1 |
| Penicillium_F29 | *Penicillium hirsutum;* *Penicillium robsamsonii*; *Penicillium thymicola* | 5e-158 | 100.00% | NR_163544.1  NR_144866.1  NR_137883.1 |
| Talaromyces_F30 | *Talaromyces stollii*; *Talaromyces funiculosus* | 5e-158 | 100.00% | NR_111781.1  MT367866.1 |

**Note:** The taxonomy of top ASVs and NE severity-associated fungal ASVs were classified according to the top hits identified by Nucleotide BLAST (blastn) in NCBI. If more than three hits showing 100% identity, only 2-3 representative species that are known to be present in the intestinal tract are shown for the sake of simplicity. If the top hit shows < 97% identity, the ASV is classified to one upper level. Identities of the top 30 ASVs were displayed.

**Table S3**. **Relative abundance (%) of top fungal taxa in the chicken ileum**

| Taxon | Score-0 | Score-1 | Score-2 | Score-5 | Score-6 | *P*-Value | FDR |  |
| --- | --- | --- | --- | --- | --- | --- | --- | --- |
| Phyla | | | | | | | | |
| Ascomycota | 73.50 ± 3.87 | 76.17 ± 2.93 | 83.37 ± 2.65 | 79.08 ± 4.45 | 81.82 ± 3.46 | 0.167 | 0.237 |  |
| Basidiomycota | 25.75 ± 3.87 | 23.08 ± 2.82 | 15.82 ± 2.62 | 17.57 ± 4.76 | 16.15 ± 3.18 | 0.092 | 0.237 |  |
| Mucoromycota | 0.74 ± 0.22 | 0.75 + 0.31 | 0.81 ± 0.13 | 3.35 ± 2.49 | 2.03 ± 0.99 | 0.237 | 0.237 |  |
| Families | | | | | | | | |
| Nectriaceae | 53.83 ± 6.73 | 55.19 ± 3.35 | 60.88 ± 4.46 | 62.99 ± 6.47 | 56.43 ± 4.78 | 0.768 | 0.805 |  |
| Wallemiaceae | 24.49 ± 3.87^a^ | 18.80 ± 2.80^a^ | 12.00 ± 2.21^b^ | 15.47 ± 4.93^ab^ | 10.95 ± 2.74^b^ | 0.019 | 0.057 |  |
| Aspergillaceae | 13.93 ± 2.53 | 13.34 ± 2.83 | 8.44 ± 1.43 | 9.79 ± 2.58 | 9.90 ± 3.17 | 0.322 | 0.460 |  |
| Pichiaceae | 0.51 ± 0.17^a^ | 2.00 ± 0.44^b^ | 6.39 ± 2.59^b^ | 1.72 ± 0.29^b^ | 5.74 ± 2.01^b^ | 3.24E-04 | 0.003 |  |
| Saccharomycetales_Incertae_sedis | 0.59 ± 0.12^a^ | 1.22 ± 0.27^b^ | 1.65 ± 0.44^b^ | 0.91 ± 0.43^ab^ | 3.74 ± 1.79^b^ | 0.015 | 0.057 |  |
| Debaryomycetaceae | 0.75 ± 0.15 | 1.57 ± 0.29 | 1.74 ± 0.41 | 1.26 ± 0.48 | 1.14 ± 0.27 | 0.137 | 0.290 |  |
| Trichocomaceae | 2.08 ± 1.16^ab^ | 0.86 ± 0.11^a^ | 0.75 ± 0.12^a^ | 0.58 ± 0.12^a^ | 2.34 ± 0.75^b^ | 0.005 | 0.034 |  |
| Trichosporonaceae | 0.26 ± 0.05^a^ | 1.12 ± 0.33^b^ | 1.65 ± 0.58^b^ | 0.95 ± 0.31^b^ | 1.70 ± 0.33^b^ | 2.75E-04 | 0.003 |  |
| Mucoraceae | 0.66 ± 0.21 | 0.71 ± 0.31 | 0.70 ± 0.13 | 3.31 ± 2.50 | 1.93 ± 0.99 | 0.541 | 0.685 |  |
| Sporidiobolaceae | 0.39 ± 0.08 | 1.27 ± 0.71 | 1.20 ± 0.68 | 0.60 ± 0.29 | 0.74 ± 0.15 | 0.687 | 0.767 |  |
| Mrakiaceae | 0.52 ± 0.22 | 1.42 ± 0.65 | 0.67 ± 0.33 | 0.21 ± 0.05 | 0.71 ± 0.33 | 0.725 | 0.795 |  |
| Sarocladiaceae | 0.55 ± 0.14 | 0.79 ± 0.22 | 1.14 ± 0.42 | 0.50 ± 0.17 | 0.43 ± 0.07 | 0.331 | 0.460 |  |
| Hydnodontaceae | 0.03 ± 0.02^a^ | 0.002 ± 0.001^a^ | 0.02 ± 0.02^a^ | 0.22 ± 0.21^ab^ | 1.33 ± 0.73^b^ | 0.002 | 0.014 |  |
| Phaffomycetaceae | 0.41 ± 0.08 | 0.57 ± 0.09 | 0.82 ± 0.25 | 0.38 ± 0.07 | 0.49 ± 0.14 | 0.776 | 0.805 |  |
| Microascaceae | 0.27 ± 0.07 | 0.12 ± 0.04 | 0.95 ± 0.80 | 0.33 ± 0.21 | 0.37 ± 0.11 | 0.205 | 0.334 |  |
| Genera | | | | | | | |  |
| Fusarium | 53.83 ± 6.73 | 55.19 ± 3.35 | 60.88 ± 4.46 | 62.98 ± 6.47 | 56.38 ± 4.79 | 0.766 | 0.821 |  |
| Wallemia | 24.49 ± 3.87^a^ | 18.80 ± 2.80^a^ | 12.00 ± 2.21^ab^ | 15.47 ± 4.93^ab^ | 10.95 ± 2.74^b^ | 0.019 | 0.082 |  |
| Aspergillus | 13.25 ± 2.49 | 9.78 ± 1.80 | 7.46 ± 1.38 | 9.44 ± 2.55 | 9.28 ± 3.14 | 0.295 | 0.483 |  |
| Pichia | 0.51 ± 0.17^a^ | 2.00 ± 0.44^b^ | 6.39 ± 2.59^b^ | 1.72 ± 0.29^b^ | 5.74 ± 2.01^b^ | 3.24E-04 | 0.005 |  |
| Candida | 0.59 ± 0.12^a^ | 1.21 ± 0.27^b^ | 1.64 ± 0.44^b^ | 0.91 ± 0.43^ab^ | 3.73 ± 1.79^b^ | 0.015 | 0.082 |  |
| Penicillium | 0.63 ± 0.14 | 3.53 ± 2.92 | 0.96 ± 0.45 | 0.31 ± 0.03 | 0.56 ± 0.10 | 0.430 | 0.600 |  |
| Meyerozyma | 0.73 ± 0.15 | 1.54 ± 0.29 | 1.71 ± 0.40 | 1.22 ± 0.48 | 1.11 ± 0.27 | 0.123 | 0.317 |  |
| Talaromyces | 2.06 ± 1.16^ab^ | 0.85 ± 0.11^a^ | 0.75 ± 0.12^a^ | 0.56 ± 0.11^a^ | 2.31 ± 0.76^b^ | 0.007 | 0.055 |  |
| Mucor | 0.66 ± 0.21 | 0.71 ± 0.31 | 0.70 ± 0.13 | 3.31 ± 2.50 | 1.93 ± 0.99 | 0.541 | 0.716 |  |
| Trichosporon | 0.20 ± 0.04^a^ | 1.09 ± 0.33^b^ | 1.61 ± 0.58^bc^ | 0.93 ± 0.31^bc^ | 1.65 ± 0.34^c^ | 1.88E-04 | 0.003 |  |
| Rhodotorula | 0.39 ± 0.08 | 1.27 ± 0.71 | 1.27 ± 0.68 | 1.20 ± 0.29 | 0.74 ± 0.15 | 0.687 | 0.780 |  |
| Tausonia | 0.52 ± 0.22 | 1.42 ± 0.65 | 0.67 ± 0.33 | 0.21 ± 0.05 | 0.71 ± 0.33 | 0.725 | 0.806 |  |
| Sarocladium | 0.55 ± 0.14 | 0.79 ± 0.22 | 1.14 ± 0.42 | 0.50 ± 0.17 | 0.43 ± 0.07 | 0.331 | 0.514 |  |
| Trechispora | 0.03 ± 0.02^ab^ | 0.003 ± 0.001^a^ | 0.02 ± 0.02^b^ | 0.22 ± 0.21^abc^ | 1.33 ± 0.73^c^ | 0.002 | 0.022 |  |
| Wickerhamomyces | 0.41 ± 0.08 | 0.56 ± 0.09 | 0.80 ± 0.25 | 0.38 ± 0.07 | 0.45 ± 0.12 | 0.754 | 0.821 |  |
| Gamsia | 0.02 ± 0.01 | 0.04 ± 0.01 | 0.83 ± 0.81 | 0.14 ± 0.09 | 0.13 ± 0.04 | 0.221 | 0.414 |  |
| Pseudotremella | 0.001 ± 0.001^a^ | 0.31 ± 0.31^a^ | 0.01 ± 0.01^a^ | 0.01 ± 0.01^ab^ | 0.04 ± 0.02^b^ | 0.026 | 0.102 |  |
| Stenocarpella | 0.27 ± 0.08 | 0.23 ± 0.13 | 0.19 ± 0.05 | 0.16 ± 0.11 | 0.33 ± 0.07 | 0.164 | 0.381 |  |
| Malassezia | 0.01 ± 0.01^a^ | 0.003 ± 0.002^a^ | 0.001 ± 0.001^a^ | 0.01 ± 0.01^a^ | 0.28 ± 0.09^b^ | 4.36 E-05 | 0.001 |  |
| Scopulariopsis | 0.25 ± 0.06 | 0.07 ± 0.04 | 0.12 ± 0.05 | 0.17 ± 0.13 | 0.24 ± 0.09 | 0.075 | 0.224 |  |
| ASVs | | | | | | | |  |
| Fusarium_F1 | 53.13 ± 6.73 | 54.22 ± 3.45 | 60.00 ± 4.47 | 62.18 ± 6.65 | 54.77 ± 4.84 | 0.742 | 0.862 |  |
| Aspergillus_vitricola_F2 | 10.67 ± 2.11^a^ | 8.21 ± 1.62^ab^ | 5.63 ± 1.34^ab^ | 8.21 ± 2.32^ab^ | 3.50 ± 0.86^b^ | 0.073 | 0.341 |  |
| Wallemia_mellicola_F3 | 6.70 ± 1.05^a^ | 5.39 ± 0.82^a^ | 3.49 ± 0.64^b^ | 4.39 ± 1.43^ab^ | 3.12 ± 0.79^b^ | 0.021 | 0.169 |  |
| Pichia_kudriavzevii_F4 | 0.51 ± 0.17^a^ | 2.00 ± 0.44^b^ | 6.29 ± 2.56^b^ | 1.71 ± 0.29^b^ | 5.60 ± 1.99^b^ | 3.44E-04 | 0.019 |  |
| Wallemia_tropicalis_F5 | 3.64 ± 0.56^a^ | 3.66 ± 0.58^a^ | 2.32 ± 0.52^ab^ | 2.90 ± 0.99^ab^ | 2.07 ± 0.58^b^ | 0.062 | 0.308 |  |
| Penicillium_F6 | 0.19 ± 0.04^a^ | 2.95 ± 2.95^b^ | 0.18 ± 0.09^abc^ | 0.03 ± 0.03^bc^ | 0.13 ± 0.05^ac^ | 0.004 | 0.077 |  |
| Wallemia_mellicola_F7 | 3.27 ± 0.53^a^ | 2.33 ± 0.32^a^ | 1.52 ± 0.27^b^ | 1.91 ± 0.60^ab^ | 1.44 ± 0.37^b^ | 0.014 | 0.148 |  |
| Wallemia_mellicola_F8 | 3.29 ± 0.55^a^ | 2.38 ± 0.37^a^ | 1.49 ± 0.30^b^ | 1.88 ± 0.60^ab^ | 1.23 ± 0.31^b^ | 0.010 | 0.122 |  |
| Wallemia_mellicola_F9 | 2.13 ± 0.33^a^ | 1.56 ± 0.25^a^ | 0.97 ± 0.17^b^ | 1.21 ± 0.39^ab^ | 0.90 ± 0.23^b^ | 0.008 | 0.112 |  |
| Meyerozyma_F10 | 0.72 ± 0.15^a^ | 1.51 ± 0.28^b^ | 1.66 ± 0.38^ab^ | 1.19 ± 0.46^ab^ | 1.07 ± 0.26^ab^ | 0.145 | 0.483 |  |
| Wallemia_mellicola_F11 | 1.83 ± 0.27^a^ | 1.43 ± 0.21^a^ | 0.90 ± 0.17^b^ | 1.33 ± 0.41^ab^ | 0.89 ± 0.22^b^ | 0.022 | 0.177 |  |
| Trichosporon_asahii_F12 | 0.18 ± 0.04^a^ | 1.03 ± 0.33^b^ | 1.57 ± 0.56^bc^ | 0.90 ± 0.31^bc^ | 1.60 ± 0.32^c^ | 1.44E-04 | 0.009 |  |
| Aspergillus_magnivesiculatus_F13 | 0.05 ± 0.01^a^ | 0.005 ± 0.003^b^ | 0.02 ± 0.01^b^ | 0.003 ± 0.003^b^ | 1.80 ± 1.79^b^ | 0.001 | 0.043 |  |
| Candida_glabrata_F14 | 0.08 ± 0.04^a^ | 0.05 ± 0.01^a^ | 0.09 ± 0.02^a^ | 0.06 ± 0.02^a^ | 3.03 ± 1.83^b^ | 0.009 | 0.122 |  |
| Fusarium_fujikuroi_F15 | 0.57 ± 0.07 | 0.87 ± 0.22 | 0.75 ± 0.10 | 0.64 ± 0.25 | 0.77 ± 0.13 | 0.592 | 0.795 |  |
| Rhodotorula_mucilaginosa_F16 | 0.31 ± 0.08 | 0.97 ± 0.49 | 1.07 ± 0.66 | 0.28 ± 0.04 | 0.58 ± 0.12 | 0.591 | 0.795 |  |
| Sarocladium_zeae_F17 | 0.48 ± 0.13 | 0.77 ± 0.22 | 1.07 ± 0.39 | 0.49 ± 0.18 | 0.41 ± 0.07 | 0.240 | 0.577 |  |
| Trechispora_F18 | 0.03 ± 0.02^a^ | 0.003 ± 0.001^a^ | 0.02 ± 0.02^a^ | 0.22 ± 0.21^ab^ | 1.33 ± 0.73^b^ | 0.002 | 0.047 |  |
| Aspergillus_F19 | 0.0007 ± 0.0007^ab^ | 0.0005 ± 0.0005^ab^ | 0.00^a^ | 0.001 ± 0.001^ab^ | 0.35 ± 0.31^b^ | 0.078 | 0.348 |  |
| Aspergillus_sydowii_F20 | 0.01 ± 0.01^a^ | 0.007 ± 0.002^a^ | 0.010 ± 0.004^a^ | 0.008 ± 0.005^ab^ | 1.59 ± 1.52^b^ | 0.045 | 0.243 |  |
| Tausonia_pullulans_F21 | 0.49 ± 0.21 | 0.87 ± 0.52 | 0.58 ± 0.29 | 0.16 ± 0.03 | 0.37 ± 0.11 | 0.806 | 0.890 |  |
| Wickerhamomyces_anomalus_F22 | 0.41 ± 0.08 | 0.56 ± 0.09 | 0.80 ± 0.25 | 0.38 ± 0.07 | 0.45 ± 0.12 | 0.754 | 0.862 |  |
| Talaromyces_proteolyticus_F23 | 0.03 ± 0.01^b^ | 0.004 ± 0.001^a^ | 0.02 ± 0.02^a^ | 0.08 ± 0.08^abc^ | 1.30 ± 0.71^c^ | 0.001 | 0.028 |  |
| Aspergillus_F24 | 0.63 ± 0.29^ac^ | 0.09 ± 0.03^b^ | 0.60 ± 0.31^abc^ | 0.11 ± 0.04^ab^ | 0.56 ± 0.16^c^ | 0.003 | 0.062 |  |
| Wallemia_tropicalis_F25 | 1.11 ± 0.21^a^ | 0.31 ± 0.10^b^ | 0.24 ± 0.08^b^ | 0.27 ± 0.16^b^ | 0.15 ± 0.07^b^ | 0.007 | 0.025 |  |
| Talaromyces_F26 | 0.54 ± 0.08 | 0.51 ± 0.07 | 0.43 ± 0.09 | 0.29 ± 0.09 | 0.47 ± 0.10 | 0.332 | 0.645 |  |
| Candida_railenensis_F27 | 0.19 ± 0.02 | 0.31 ± 0.06 | 0.83 ± 0.32 | 0.31 ± 0.13 | 0.32 ± 0.12 | 0.397 | 0.691 |  |
| Gamsia_aggregata_F28 | 0.02 ± 0.01 | 0.04 ± 0.01 | 0.83 ± 0.81 | 0.13 ± 0.04 | 0.12 ± 0.04 | 0.198 | 0.556 |  |
| Penicillium_F29 | 0.35 ± 0.15 | 0.50 ± 0.19^b^ | 0.26 ± 0.08 | 0.23 ± 0.05 | 0.34 ± 0.08 | 0.409 | 0.699 |  |
| Talaromyces_F30 | 1.28 ± 1.09^a^ | 0.16 ± 0.02^ab^ | 0.15 ± 0.02^ab^ | 0.09 ± 0.03^b^ | 0.20 ± 0.03^a^ | 0.051 | 0.260 |  |

**Note**: Relative abundances of (%) of all phyla, top 15 families, top 20 genera, and top 30 amplicon sequence variants (ASVs) of the ileal mycobiota in chickens with varying NE lesion scores were indicated as means and SEM. Statistical significance was determined using Kruskal-Wallis test followed by pairwise Mann–Whitney U test. False discovery rate (FDR) was controlled by the Benjamini-Hochberg procedure. *P <* 0.05 was considered significant.
